# The Mining of Candidate Genes Involved in the Camphor Biosynthesis Pathway of *Cinnamomum camphora*

**DOI:** 10.3390/plants14070991

**Published:** 2025-03-21

**Authors:** Yan Yang, Shengcai Zhou, Mingyang Ni, Yuting Zhang, Shixiong Lin, Junhong Zhang, Zaikang Tong

**Affiliations:** 1 Key Laboratory for Development and Utilization of Forest Food Resources, Zhejiang A&F University, Hangzhou 311300, China; yy632379@sina.com (Y.Y.); 15158063607@163.com (M.N.); zhangyutingly@126.com (Y.Z.); ling854200699@163.com (S.L.); zktong@zafu.edu.cn (Z.T.); 2 Zhejiang Key Laboratory of Forest Genetics and Breeding, Zhejiang A&F University, Hangzhou 311300, China; 3 College of Forestry Science and Technology, Lishui Vocational and Technical College, Lishui 323000, China; zhoushengcai@lszjy.edu.cn

**Keywords:** *Cinnamomum camphora*, chemotypes, transcriptomes, monoterpene, TPS

## Abstract

*Cinnamomum camphora* is widely cultivated for its camphor in essential oil (EO), which is used in pharmaceutical industries. However, the candidate genes for the camphor biosynthesis pathway are unknown. Gas chromatography–mass spectrometry (GC-MS) was used to identify differences in the composition of camphor- and linalool-type camphor EOs and in conjunction with transcriptional analysis to identify terpene biosynthesis-related genes. The GC-MS analysis of *C. camphora* revealed 67 chemical components, including 32 monoterpenes and 35 sesquiterpenes, with camphor-type leaves dominated by camphor and linalool-type leaves by linalool. Transcriptome analysis revealed 6499 differentially expressed genes (DEGs) between camphor- and linalool-type *C. camphora*, with 4244 upregulated and 2255 downregulated in the camphor-type. GO enrichment highlighted DEGs involved in monoterpene biosynthesis, cell wall organization, and membrane-related processes. KEGG analysis identified pathways such as monoterpenoid, diterpenoid, and phenylpropanoid biosynthesis as significantly enriched. Furthermore, DEGs encoding *TPS*, dehydrogenases, and transcription factors, which might contribute to the terpenoid diversity in *C. camphora*, were identified. Twenty-one candidate genes involved in the camphor biosynthesis pathway were identified, providing a foundation for further elucidating the genetic mechanisms underlying camphor production in *C. camphora.*

## 1. Introduction

Chemotypes are organisms categorized within the same species, subspecies, or varieties, with differences in the composition of secondary metabolites [1]. Having high commercial values, these secondary metabolites are exploited largely as flavors, fragrances, and pharmaceuticals [2]. The chemotype diversity of the EO was characterized in many plant species, including *Trachyspermum ammi* [3], *Psidium guajava* [4], *Cinnamomum burmanni* [5], *Cinnamomum camphora* [6], etc. The chemotype of plant species has traditionally been defined as its profile of natural products, and the genotype has been defined as its genetic constitution or DNA sequence [1]. However, minor genetic changes with no significant effect on morphology or anatomy may trigger large changes in the chemical phenotype [7].

*C. camphora*, a representative species in the Lauraceae family, is an evergreen aromatic tree indigenous to Southern China and South Korea [8], which is listed as a Class II protective tree species in China [9]. *C. camphora* has become of increasing importance as a resource of food additives and as raw materials for the cosmetic and pharmaceutical industries. Previous studies showed that the leaves, stems, roots, and flowers are abundant in EOs, most of which are terpenoids, especially for monoterpenes and sesquiterpenes [10]. Five chemotypes, including the isoborneol-type, camphora-type, cineole-type, linalool-type, and borneol-type, have been identified in *C. camphora* [6]. Linalool is a colorless liquid belonging to linear monoterpenes. Linalool shows significant therapeutic potential in the treatment of arthritis [11]. The content and chemical compositions of EO from linalool-type *C. camphora* are dependent on organ and/or tissues and season [12]. Camphor, a bicyclic saturated terpene ketone, is structurally similar to borneol and is also widely used in traditional Chinese medicine [13]. Natural camphor has medicinal and catalyst properties. For example, the cool and aromatic camphor can be used to alleviate skin itching and irritation [14].

Terpenoids (or terpenes), including 55,000 members, are the largest class of structurally diverse metabolites in all forms of life, and the family of terpene or terpenoid natural products represents the epitome of molecular biodiversity [15]. Terpenoids are classified into monoterpene (C10), sesquiterpenes (C15), diterpenes (C20), triterpenes (C30), tetraterpenes (C40), and polyterpenes with more than eight 5C units [16]. Most of the monoterpenes are produced by the modification of GPP, which are processed in four steps from the generation of the terpenoid building units (IPP and DMAPP) [17]. For example, DMAPP initially generates GPP, which is subsequently cyclized and hydrolyzed to borneol through borneol synthases; then, the borneol can be oxidized to camphor by the borneol dehydrogenase (BDH) [18]. BDH is a subfamily of short-chain dehydrogenases/reductase (SDR), one of the largest and oldest protein superfamilies [19], with 449 families in the nomenclature [20]. The SDR superfamily is widespread, having members from all kingdoms and with members typically showing 20–30% residue identity [21], increasing the difficulty of identification.

The wealth of terpene carbon skeletons can be attributed to an enzyme class known as terpene synthases (TPS). TPS, as key enzymes, use several steps of cyclization and oxidation to catalyze the precursors of each terpenoid family, triggering the formation of a simple or mixed compound of reaction products of terpenoid metabolites [22]. Many isoprene, monoterpene, sesquiterpene, or diterpene synthases of different plant species form TPS subfamilies (TPSa–g), with some plant lineages having a majority of their TPS genes in one or two clades [23]. For example, TPS-d is specific to gymnosperm [24], while TPS-a, TPS-b, and TPS-g are specific to angiosperm plants [23]. The TPS-b subfamily encodes either monoterpene synthases or isoprene synthases [23], and further study supports the evolution of isoprene synthases from ocimene synthases [25]. Genes in the TPS-a subfamily are mainly sesquiterpene synthases, which appear to be highly divergent in all seed plants [23]. The TPS-c clade is located at the base of the phylogeny tree, and the members are copalyl synthase (CPS) or diterpene synthases. TPS-e and TPS-f are closely related to the TPS-c clade, which are mainly kaurene synthase (KS) and diterpene synthases [23]. The TPS-g clade, closely related to TPS-b, contributes to acyclic monoterpene synthesis, such as myrcene and ocimene in snapdragon [26]. The TPS-h subfamily is specific to *Selaginella moellendorffii*, a putative bifunctional TPS clade, which might have evolved from CPS/KS and is involved in specialized metabolism in lycophytes [23].

Accumulating evidence shows that transcription factors play essential regulatory roles in secondary metabolism pathways. For example, CmWRKY41 targets *CmHMGR2* and *CmFPPS2* to positively regulate sesquiterpene biosynthesis in chrysanthemums [27]. Hong reported a basic helix–loop–helix transcription factor, MYC2, that directly binds to the promoters of the sesquiterpene synthase genes *TPS21* and *TPS11* and activates their expression in *Arabidopsis* [28]. Three *NAC* genes regulate *AaTPS1* transcription, which, in turn, controls monoterpene production in kiwifruit [29]. Wang showed that a novel *MsYABBY5*, which encodes a transcription factor, is probably a repressor of secondary metabolism in *Mentha spicata* [2].

Next-generation sequencing (NGS) techniques are valuable tools that have been used to identify and characterize secondary metabolism genes and their pathways. For example, in order to identify putative genes involved in papaverine biosynthesis, comparative transcriptome analysis between a high papaverine mutant (pap1) and a normal cultivar (BR086) was conducted in *Papaver somniferum* [30]. Transcriptome sequencing was also used to identify putative genes involved in thymol and other monoterpene biosynthesis pathways in *T. ammi*; thus, differentially expressed unigenes encoding dehydrogenases, transcription factors, and cytochrome P450s, which might be associated with terpenoid diversity in *T. ammi*, were identified [31]. To decipher the terpenoid biosynthesis of the major monoterpenes in two chemotypes, the leaf transcriptomes of linalool- and borneol-type chemotypes of *C. camphora* were analyzed using RNA sequencing. A total of 2863 unigenes were identified to be differentially expressed between the two chemotypes, and 67 candidate unigenes were identified to be involved in terpenoid biosynthesis in *C. camphora* [32]. In the present study, the terpenoid components of leaf EO were compared between camphor- and linalool-type of *C. camphora* using gas chromatography–mass spectrometry (GC-MS), Subsequently, two groups of transcriptome data with three replicates were analyzed using RNA-Seq.

## 2. Results

### 2.1. The Composition of Leaf Extracts from Two Chemotypes of C. camphora

**Figure 1:**
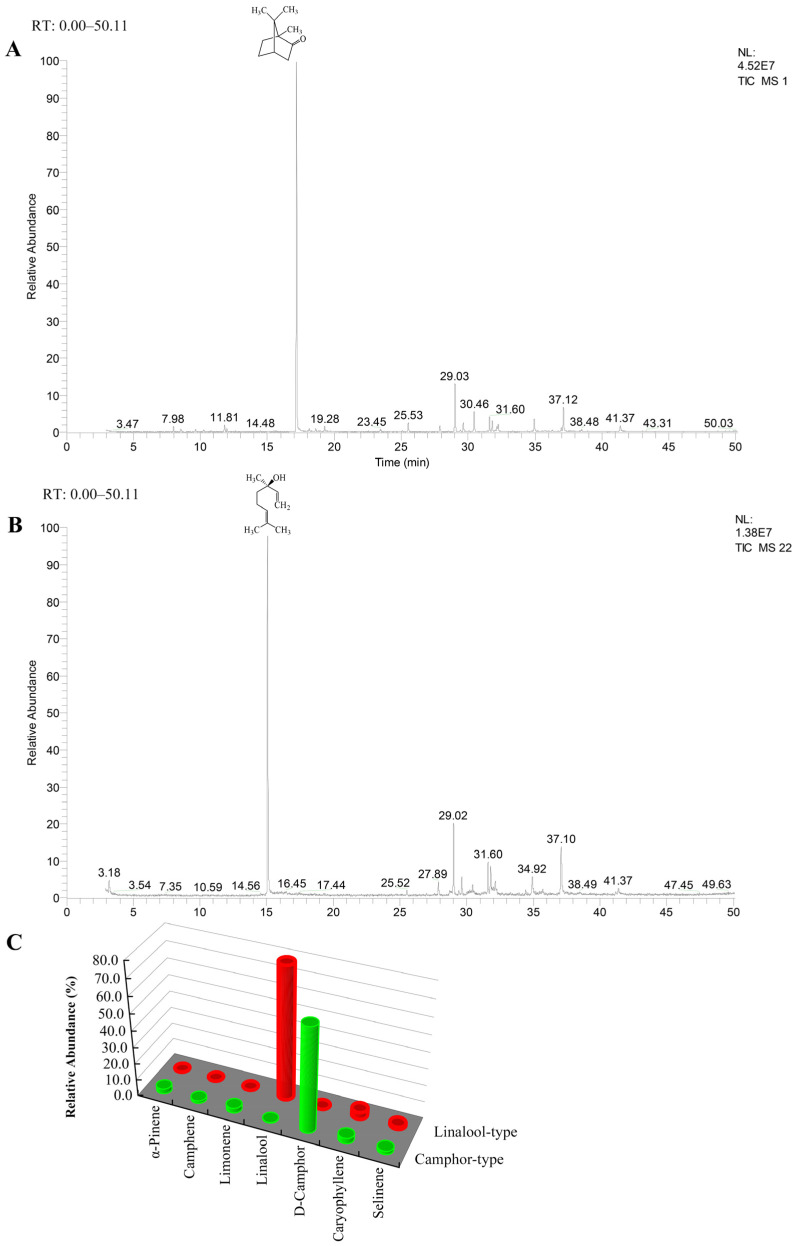
GC/MS profile of leaf EO in *C. camphora*. (**A**) Camphor type of *C. camphora*, (**B**) linalool type of *C. camphora*. (**C**) Relative quantification of main components for EO in two chemotypes.

A total of 58 chemical components were detected in the EO of camphor-type leaves, including 25 monoterpenes and 33 sesquiterpenes, and camphor was the main monoterpene, accounting for 62.85 ± 5.50% (Figure 1A, Table S1). A total of 61 chemical components were observed in the leaves of linalool-type *C. camphora*, including 28 monoterpenes and 31 sesquiterpenes, and linalool was the main monoterpene, accounting for 79.44 ± 5.53% (Figure 1B, Table S1). In total, 67 chemical components were identified from two chemotypes of *C. camphora*, including 32 monoterpenes and 35 sesquiterpenes, among which 53 components were common to both chemotypes (Table S1). The major components of the two chemotypes are shown in Figure 1C, highlighting the distinct chemical composition of EO between the two chemotypes.

### 2.2. The Statistics of RNA Sequencing in Two Chemotypes of C. camphora

In an attempt to compare the biosynthetic pathways of EO in two chemotypes of *C. camphora*, we obtained the transcriptome data from both chemotypes using RNA-seq. More than 250 million clean reads were obtained from six RNA libraries, and the mapping rates to the reference genome (*Cinnamomum kanehirae*) were superior, with a range from 84.89% to 86.98% (Table S2). Further analysis revealed that the majority of the mapped reads were in exon regions, accounting for more than 88% of the total, and a small proportion of reads aligned in intronic and intergenic regions, respectively (Figure S1). In addition, correlation and PCA of gene profiles from six samples demonstrated high reproducibility within each chemotype (Figure S2).

### 2.3. Characterization of the Differentially Expressed Genes

**Figure 2:**
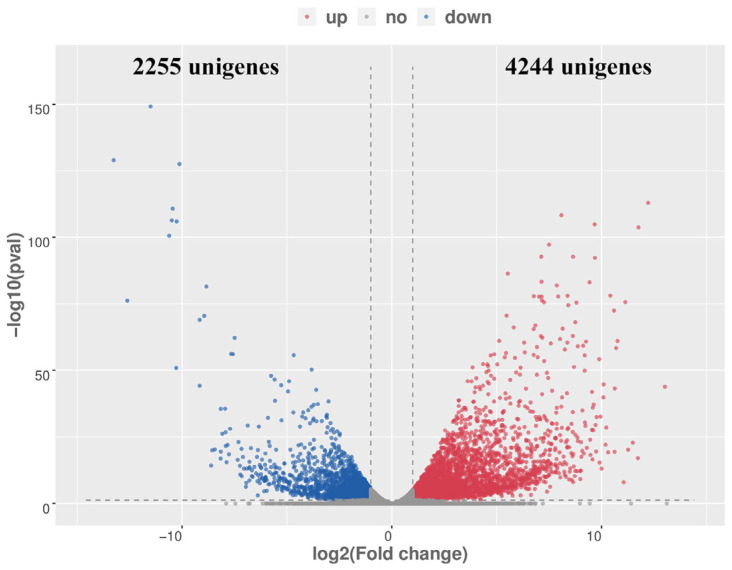
Analysis of differentially expressed genes (DEGs) between two chemotypes of *C. camphora*. The dotted vertical line indicates log2 (fold change) = ±1, and dashed horizontal lines indicate log10 (*p*-value) ≈ 1.301 (*p*-value = 0.05).

In total, the expression patterns of 24,946 genes were compared between two chemotypes, revealing 6499 genes that were differentially expressed, including 4244 upregulated and 2255 downregulated genes in the camphor type compared to the linalool type of *C. camphora* (Figure 2, Table S3). GO enrichment analysis of differentially expressed genes (DEGs) revealed that, in the biological process category, the enriched terms included ‘cell wall organization’, ‘metabolic processes of monoterpene biosynthesis’, ‘geranyl diphosphate metabolism’, ‘lipid biosynthesis’, ‘cellulose biosynthesis’, ‘xylan biosynthesis’, and ‘flavonoid biosynthesis’. In the molecular function category, the enriched terms included ‘oxidoreductase activity, oxidizing metal ions’, ‘geranyl diphosphate metabolic process’, and ‘polyamine oxidase activity’. In the cellular component category, the most abundant genes were associated with ‘plasma membrane’, ‘integral component of membrane’, and ‘extracellular region’ (Table S4 and Figure S3A). KEGG enrichment analysis of DEGs identified 29 pathways, including ‘Monoterpenoid biosynthesis’, ‘Diterpenoid biosynthesis’, ‘Sesquiterpenoid and triterpenoid biosynthesis’, ‘Benzoxazinoid biosynthesis’, ‘Pentose and glucuronate interconversions’, ‘Phenylpropanoid biosynthesis’, and ‘Galactose metabolism’ significantly enriched pathways (*p* < 0.01) (Table S5 and Figure S3B).

### 2.4. Identification of Genes Involved in Terpenoid Biosynthesis

**Figure 3:**
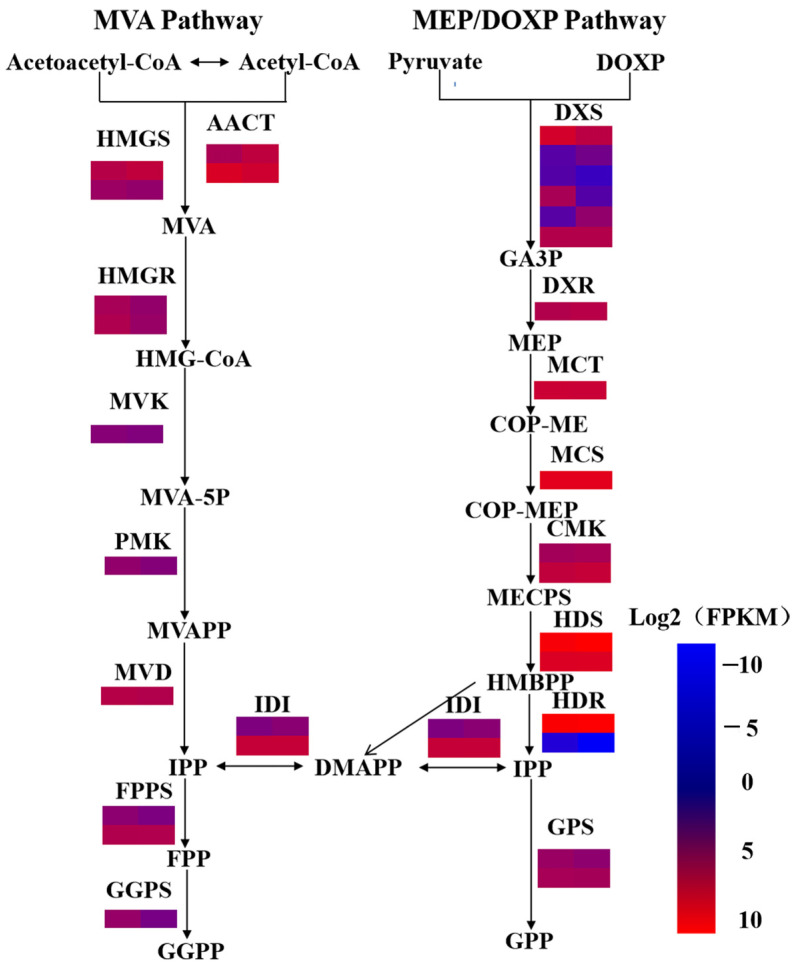
Expression patterns of genes involved in the terpenoid backbone biosynthesis pathways of two chemotypes of *C. camphora*. Heatmap showing the log2 (FPKM) expression values of genes encoding enzymes involved in the terpenoid backbone biosynthesis pathways in camphor-type and linalool-type *C. camphora*. The expression pattern of each unigene is shown within two-column grids, with the left column representing the camphor type and the right one representing the linalool type. DMAPP, dimethylallyl diphosphate; *DXR*, 1-deoxy-D xylulose-5-phosphate reductoisomerase; *DXS*, 1-deoxy-D-xylulose-5-phosphate synthase; FPP, farnesyl diphosphate; *FPPS*, FPP synthase; *GPS*, geranyl diphosphate synthase; GGPP, geranylgeranyl pyrophosphate; *GGPS*, GGPP synthase; GPP, geranyl pyrophosphate; *AACT*, acetyl-CoA C-acetyltransferase; *HMGS*, hydroxymethylglutaryl-CoA synthase; *HMGR*, hydroxymethylglutaryl-CoA reductase (NADPH); *MVK*, mevalonate kinase; *PMK*, phosphomevalonate kinase; *MVD*, diphosphomevalonate decarboxylase; *MCT*, 2-C-methyl-D-erythritol 4-phosphate cytidylyltransferase; *CMK*, 4-diphosphocytidyl-2-C-methyl D-erythritol kinase; *MCS*, 2-C-methyl-D-erythritol 2,4-cyclodiphosphate synthase; *HDS*, (E)-4-hydroxy-3-methylbut-2-enyl-diphosphate synthase; *HDR*, 4-hydroxy-3-methylbut-2-en-1-yl diphosphate reductase; *IDI*, isopentenyl-diphosphate Delta-isomerase.

A total of 843 genes were annotated as related to secondary metabolism, encompassing 19 KEGG pathways with 156 KO entries, of which 284 genes were differentially and significantly expressed between two chemotypes, with 83 KO entries (Table S6). In terpenoid backbone biosynthesis, a key intermediate pathway for terpenoid synthesis, 65 genes encoding the processing enzymes were identified in the transcriptome data. The data showed that most enzymes were encoded by multiple gene copies, some of which were differentially expressed between the two chemotypes of *C. camphora*. The highest number of genes was assigned to *DXS* (six genes), followed by *AACT*, *HMGS*, *HMGR*, *CMK*, *HDS*, and *IDI* with two genes, while the remaining genes were present as single copies (Figure 3, Table S7). Most genes encoding key enzymes in the terpenoid backbone pathway were not differentially expressed between two chemotypes, except for four *DXS* genes, one *HMGR* gene, and one *GPPS* gene (Figure 3).

### 2.5. Analysis of TPS Families in C. camphora

A total of 83 *CcTPS* genes were predicted and annotated in *C. camphora* by searching two Pfam domains (PF03936 and PF01397), designated as *CcTPS1* to *CcTPS83* (Table S8). The length of *CcTPS* genes ranged from 213 to 1615 amino acids, with an average length of 569 amino acids. The predicted molecular weight ranged from 23,947.9 to 184,420.9, with an average of 65,551.2; the pI value ranged from 4.57 to 8.94, with a mean value of 5.64. Among the 83 *CcTPS* genes, 42 members were annotated as monoterpenoid synthesis enzymes, of which 27 monoterpenoid-related genes were differentially expressed between the two chemotypes and 17 genes were upregulated in the camphor type compared to the linalool type.

**Figure 4:**
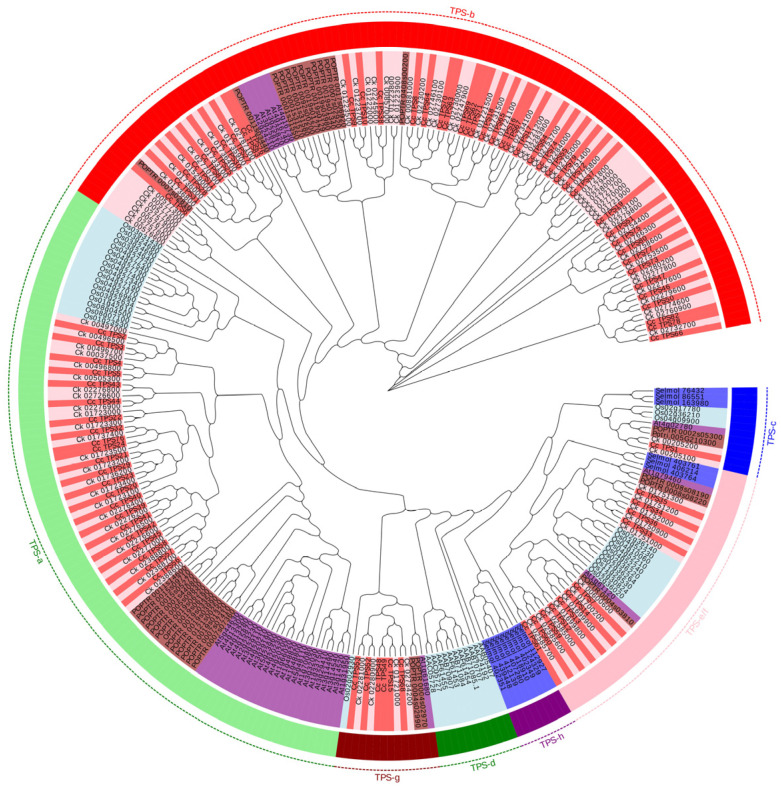
Phylogenetic placements of the TPS genes in *C. camphora*. The phylogenetic tree was constructed using predicted or characterized TPS genes from seven sequenced land plant genomes. Cyan in the outer lap represents the TPS-a subfamily, red represents the TPS-b subfamily, dark blue represents the TPS-c subfamily, green represents the TPS-d subfamily, pink represents the TPS-e/f subfamily, crimson represents the TPS-g subfamily, and violet represents the TPS-h subfamily. The inner lap in red represents the *TPS* genes in *C. camphora*, pink represents the *TPS* genes in *C. kanehirae*, purple represents the *TPS* genes in *Arabidopsis thaliana*, light blue represents the *TPS* genes in *Oryza sativa*, purplish blue represents the *TPS* genes in *S. moellendorffii*, and brown represents the *TPS* genes in *P. trichocarpa.* The *TPS* genes in *A. grandis* belong only to the TPS-d subfamily.

Phylogenetic analysis of *TPS* from seven species classified *CcTPS* genes among five of seven *TPS* gene subfamilies previously described in land plants [23]. Similar to *C. kanehirae*, the CcTPS-a and CcTPS-b subfamilies contained the most abundant members, with 22 and 43 genes, respectively (Table S9 and Figure 4), followed by CcTPS-e/f and CcTPS-g, which contained 11 and six members, respectively. *Cc_TPS15*, *Cc_TPS48*, *Cc_TPS49*, *Cc_TPS52*, *Cc_TPS53*, and *Cc_TPS68* were clustered into the CcTPS-g subfamily. In *A. thaliana*, 33 *TPS* genes were predicted, and TPS-a contained the most members, with 23 genes. Interestingly, TPS-b was absent in *O. sativa*, while TPS-a contained 17 members, followed by TPS-e/f, with 10 members. Although only 14 members were predicted in the *S. moellendorffii* genome, TPS-h was unique to land plants. The TPS-d clade was specific to *Abies grandis*, with 11 members. In addition, the TPS-a and TPS-b subfamily contained the same number of members in *Populus trichocarpa* (Table S9 and Figure 4).

### 2.6. Analysis of SDR Families in C. camphora

**Figure 5:**
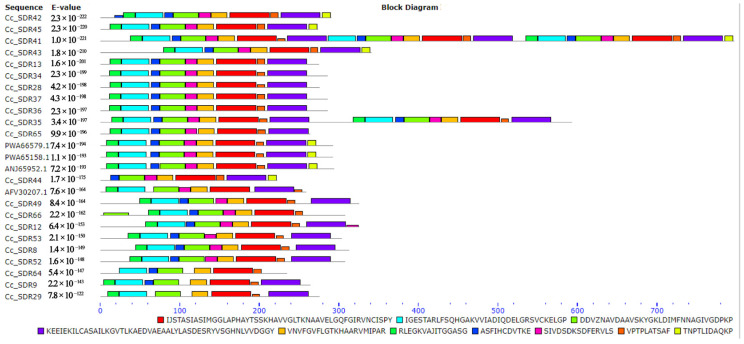
The motif analysis of the *CcSDR* genes in *C. camphora*.

A total of 75 *SDR* genes were predicted in *C. camphora*, designated as *CcSDR1* to *CcSDR75* (Table S10). The length of *CcSDR* genes ranged from 669 to 3126 bp. These genes were classified into 23 subfamilies, and 21 members belonging to the SDR110C subfamily were selected for further analysis. Conserved motif analysis for 21 *CcSDR* genes identified ten motifs (Figure 5). Four *SDR* genes, including *CcSDR41*, *CcSDR42*, *CcSDR43*, and *CcSDR45*, showed the highest similarity to four referenced *BDH* genes, and therefore, these four *CcSDR* genes were selected as *CcBDH* candidates.

### 2.7. Analysis of Transcription Factor Genes Related to Terpenoid Biosynthesis

**Figure 6:**
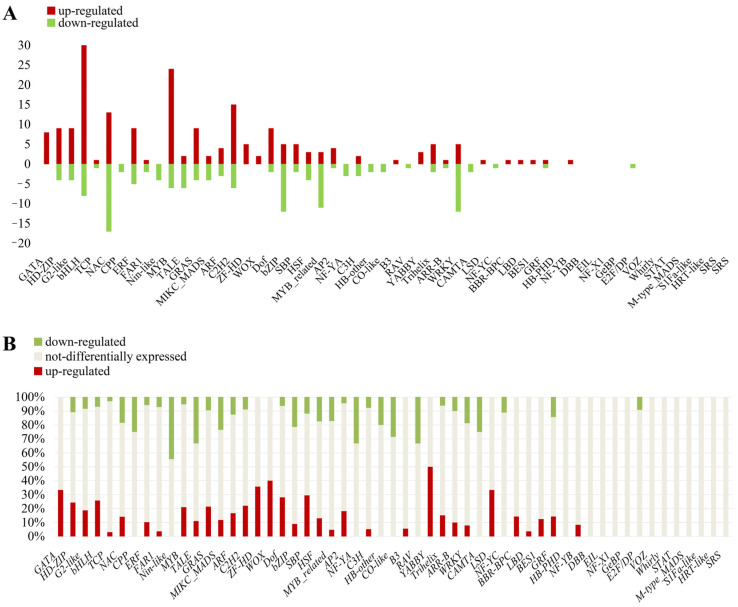
The identification of transcription factor genes in *C. camphora*. (**A**) Percentage of identified TFs families. (**B**) Percentage of differentially expressed TFs families.

The BLASTx search against the Plant TF database (V4.0) under the default parameters identified 1339 putative transcription factors (TF) from 55 families, showing high homology with TFs from 165 plant species. In the present study, the dominant families were bHLH, MYB, NAC, and ERF (Figure 6A), each accounting for more than 5% of the total. A total of 373 genes from 45 TF families showed differential expression between the two chemotypes, including bHLH (40 genes), MYB (39 genes), NAC (32 genes), C2H2 (24 genes), and WRKY (20 genes) (Figure 6A). Among these differentially expressed TF genes, 229 were upregulated, while 144 were downregulated in the camphor type compared to the linalool type. All ZF-HD (five genes), TCP (four genes), YABBY (four genes), and WOX (three genes) were upregulated in the camphor type, whereas all MIKC_MADS (five genes), Nin-like (four genes), NF-YA (four genes), and CPP (three genes) were downregulated in the camphor type. However, members of these big TF families, such as bHLH, MYB, NAC, C2H2, and WRKY, showed distinct expression levels between the two chemotypes. For example, 30 of 40 *bHLH* genes, 29 of 39 *MYB* genes, and 18 of 24 *C2H2* members were upregulated in the camphor type, while 15 of 20 *WRKY* and 17 of 32 *NAC* members were upregulated in the linalool type (Figure 6B).

### 2.8. RT-qPCR Confirmation of RNA-Seq Data

**Figure 7:**
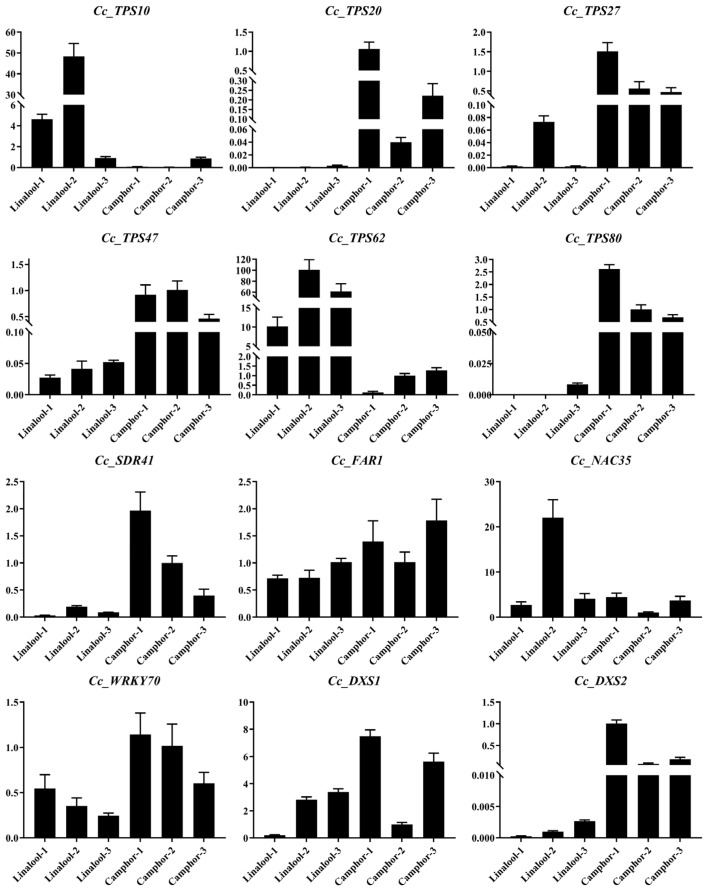
The RT-qPCR validation of candidate genes. *Actin* served as the reference gene. All the experiments were performed with three replicates, and the results are presented as mean ± SD.

To validate the expression patterns of terpenoid-related genes obtained using RNA-Seq analysis, RT-qPCR was performed to compare the expression levels of 12 genes between the two chemotypes (Figure 7). The expression patterns of all selected genes obtained from RT-qPCR were consistent with those deduced from their FPKM values in the RNA-seq data (Figure S5A). Correlation analysis showed that the RT-qPCR results were significantly correlated with RNA-seq data (*r* = 0.620, *p* = 0.031, Figure S5B). The RT-qPCR results confirmed the reliability of the transcriptomic profiling, although the fold change of some genes differed between the RT-qPCR and RNA-seq data. Furthermore, to evaluate the expression levels of these 12 genes in other individuals, we selected two additional trees from each chemotype to analyze their expression patterns. The results showed that nine genes showed similar expression patterns in each chemotype, while three genes, including *Cc_FAR1*, *Cc_NAC35*, and *Cc_DXS1*, exhibited inconsistent expression patterns within the chemotype (Figure 7). For example, *CcTPS10* and *CcTPS62* were upregulated in the linalool type, while the other four *CcTPS* genes were upregulated in the camphor type.

## 3. Discussion

Previous studies have shown that monoterpenes are the main components of leaf EO in many species of the *Cinnamomum* genus, including *C. kanehirae*, *Cinnamomum osmophloeum*, and *C. camphora* [6]. Based on the most abundant monoterpenes, *C. camphora* has been classified into five chemotypes [6]. In this study, two chemotypes of *C. camphora* were identified, the main chemical components being monoterpenes, specifically camphor and linalool, respectively. Which genes contribute to the distinct EO in the two chemotypes? Two transcriptomes were used to elucidate the potential genes participating in the specific monoterpene biosynthesis.

Previous studies have shown that both the chloroplastic 2-C-methyl-D-erythritol 4-phosphate (MEP) pathway and the cytosolic mevalonate (MVA) pathway produce the five-carbon building blocks, isopentenyl diphosphate (IPP) and dimethylallyl diphosphate (DMAPP) [22]. In our study, 65 genes encoding processing enzymes were identified, of which only four *DXS*, one *HMGR*, and *GPPS* were differentially expressed between two chemotypes (Figure 3). Previous studies have shown that HMGR and DXS are two rate-limiting enzymes in the MVA [33] and MEP pathways [34], respectively. This indicated that *DXS* and *HMGR*, as rate-limiting enzyme genes, might contribute to the distinct chemical components in the two chemotypes. Transcriptome data revealed that two *DXS* genes were upregulated in the linalool type, and two *DXS* were upregulated in the camphor type (Figure 3). RT-qPCR results validated that *Cc_DXS1* and *Cc_DXS2* were upregulated in the sequenced sample (Camphor 1) relative to the Linalool 1 sample (Figure S5), and further RT-qPCR analysis showed that *Cc_DXS2* was upregulated in all three camphor-type individuals, while *Cc_DXS1* was inconsistently expressed in the camphor type, such as in Camphor 2 (Figure 7). Therefore, *Cc_DXS2* might play a role in camphor synthesis.

Several studies have suggested that gymnosperm and angiosperm monoterpene and sesquiterpene synthases evolved from an ancestral diterpene synthase [35]. The length of plant TPSs varies, typically ranging from 600 to 900 amino acids. Variations in the three-domain structure of the TPS family might be attributed to the loss of a particular domain, such as the KS- or CPS-type domains, during the evolution of different TPS lineages [23]. In this study, 27 monoterpenoid-related genes were differentially expressed between the two chemotypes, which was consistent with the distinct chemical components of EO between the two chemotypes (Figure 1). It suggests that the different expression of *TPS* genes in two chemotypes may contribute to their different terpenoid compositions.

Previous studies have shown that camphor, an anti-inflammatory compound [36], is a bicyclic saturated monoterpenoid catalyzed by the TPS-b subfamily [13,23]. In the present study, six *TPS* genes annotated as linalool synthases showed no significant expression difference between the two chemotypes; this suggests that these *TPS* genes might not participate in the linalool synthesis. *CcTPS27* was aligned with trans-ocimene synthase and was significantly upregulated in the camphor type (Figure 7), consistent with the higher ocimene content in the camphor type (Table S1). CcTPS47 and CcTPS80 were annotated as geraniol synthases and showed upregulated expression in the camphor type, although the geraniol was not detected in the camphor type. Several studies have shown that the specific biochemical roles of individual TPS members should not be predicted based on sequence similarity, as changes in only a few amino acids may lead to significant alterations in the terpenoid profiles of a given TPS enzyme [26,37]. Furthermore, many *TPS* genes encode multi-product enzymes, which often catalyze mixtures of the same compounds in differing proportions [24]. However, it remains unclear whether specific protein features facilitate the formation of multiple products [38].

In addition, terpenoid biosynthesis can be regulated by alternative splicing of key *TPS* genes [39]. A similar phenomenon was observed in *Torreya grandis*, where the inconsistency between β-sitosterol content and the expression level of the enzyme gene *TgSMT2* suggests that post-transcriptional regulation may be important for the enzymatic activity of TgSMT2 [40]. Therefore, *CcTPS27*, *CcTPS47*, and *CcTPS80* might contribute to the biosynthesis of ocimene and other monoterpenoids in the camphor type.

*CcTPS10* and *CcTPS62* were aligned with alpha-terpineol synthase and were upregulated in three linalool-type individuals (Figure 7). Consistently, although relatively low abundance was detected in the linalool type, the terpineol content was undetected in the camphor type. Therefore, *CcTPS10* and *CcTPS62* might contribute to the biosynthesis of terpineol and other monoterpenoids in the linalool type.

Conserved motif analysis of the 21 *CcSDR* genes revealed that *CcSDR41*, *CcSDR42*, *CcSDR43*, and *CcSDR45* might be *CcBDH* candidates (Figure 5). Further expression analysis showed that *CcSDR41* was upregulated in three camphor-type individuals (Figure 7). In *Lavandula*, *LiBDH* was specifically expressed in glandular trichomes of mature flowers and was reported to specifically convert borneol to camphor [41].

Previous studies have suggested that terpenoid synthesis is a complex process, except that the related enzymes and transcription factors (TFs) play essential roles in the terpenoid synthesis process [42,43]. For example, the transcription factor WRKY45 plays a central role in the biosynthesis of diterpenoid phytoalexins during rice–Magnaporthe oryzae interactions. TFs usually activate or repress the promoters of *TPS* genes to control their expression and regulate terpenoid accumulation. For example, He et al. demonstrated that three *WRKY* genes, including *WRKY61*, *WRKY28*, and *WRKY40*, might coordinately trans-activate the NeoD promoter in *Amomum villosum* [42]. In this study, 20 *WRKY* genes were differentially expressed between the two chemotypes, with 15 members upregulated in the linalool type (Figure 6). We found that *Cc_WRKY70* showed relatively higher expression levels in the camphor type (Figure S5 and Figure 7). Further analysis showed that Cc_WRKY70 contains a complete WRKY domain, including the nuclear localization sequence (NLS) located at the N-terminal of the conserved WRKY domain. The predicted Cc_WRKY70 protein consists of a characteristic WRKY domain, containing both the oligopeptide ‘WRKYGQK’ and a putative zinc finger motif ‘C-X7-CX23-H-X-C’ (Figure S4). This suggests that *Cc_WRKY70* has biological activity, as indicated in previous studies [44,45], which might be involved in the synthesis and regulation of camphor.

In addition, 32 *NAC* genes were differentially expressed between the two chemotypes, with 15 members being upregulated in the camphor type (Figure 6). Transcriptome data and RT-qPCR results showed that *Cc_NAC35* was upregulated in sequenced individuals of the camphor type, while further RT-qPCR results revealed that *Cc_NAC35* was inconsistently upregulated in the camphor type (Figure S5). This suggested that *Cc_NAC35* might be unrelated to camphor synthesis.

In conclusion, our study identified a set of candidate genes whose differential expression may be responsible for the production of two chemotypes in *C. camphora*. However, it is important to acknowledge that the identification of these candidate genes was based solely on differential expression analysis, and their functional roles in terpenoid biosynthesis remained to be experimentally validated, which was a shortcoming of this study. Functional validation through techniques such as gene knockout, overexpression, or enzymatic assays will be essential to confirm their specific contributions to camphor and linalool biosynthesis. Additionally, our study did not account for potential epigenetic modifications, which could significantly influence gene expression and terpenoid accumulation. Epigenetic regulation, such as DNA methylation and histone modifications, may play a critical role in the observed differences in terpenoid profiles between the two chemotypes. Future studies should incorporate epigenetic analyses to provide a more comprehensive understanding of the regulatory mechanisms underlying terpenoid biosynthesis. Furthermore, exploring the interactions between transcription factors, such as *Cc_WRKY70*, and their target genes, as well as investigating post-transcriptional and post-translational regulatory mechanisms, will be crucial for elucidating the complex network governing terpenoid biosynthesis in *C. camphora.* Addressing these limitations in future research will enhance our understanding of the molecular basis of chemotype differentiation and provide valuable insights into the metabolic engineering of terpenoids in this economically important species.

## 4. Materials and Methods

### 4.1. Plant Materials

For chemical analysis and RNA sequencing, leaves of camphora-type and linalool-type *C. camphora* were collected from trees grown at the nursing base of Zhejiang A&F University (30.256632° N, 119.728224° E). All samples were collected from healthy and mature camphor trees, and the sampling time was uniformly at 11 am to minimize the influence of circadian rhythms on gene expression. The sampling site was the young leaves of the current year because this site is the main place for secondary metabolite synthesis. The two chemotypes of *C. camphora* were extremely close geographically and grew under the same environmental conditions. In the summer, the compositions of their leaf extracts are listed in Table S1. All the samples used for RNA exaction were frozen in liquid nitrogen immediately and stored at −80 °C. Three biological repeats were conducted.

### 4.2. Phytochemical Analysis

The analysis of EO from leaf of two chemotypes was carried out using GC/MS. The fresh, healthy leaves (100 g) of the tree were powdered and hydrodistilled and extracted with a clevenger apparatus for 3 h until no further increase in oil volume. The chemical composition of EOs was analyzed on a Trace GC/ISQ gas chromatograph–mass spectrometer (Thermo Fisher, Waltham, MA, USA) equipped with a TM-5MS silica capillary column (30 m × 0.25 mm × 0.25 μm). Helium was used as the carrier gas at a constant flow rate of 1.0 mL/min. The GC temperature program was set as follows: initial temperature of 50 °C held for 2 min, followed by a gradient increase to 180 °C at a rate of 3 °C/min, holding at 180 °C for 2 min, then a further gradient increase to 220 °C at a rate of 8 °C/min, and finally holding at 220 °C for 5 min, with a total run time of 50 min. Alkanes were used as reference points for calculating relative retention indices. The injection volume was 0.1 μL. The MS operating parameters were configured as follows: electron ionization (EI) mode at 70 eV; ion source temperature of 200 °C; transfer line temperature of 250 °C; and a scan mass range of 40–500 *m*/*z*. Peaks were identified by comparing their retention times with those of known standards analyzed under the same conditions. A library search was conducted using the NIST08 library, and the most closely matched results were selected. Finally, the relative content of each peak was determined using the peak area normalization method.

### 4.3. RNA Sequencing and Transcriptome Analysis

Total RNA was extracted using Trizol reagent (Invitrogen, Carlsbad, CA, USA) according to the manufacturer’s procedure. The total RNA quantity and purity were assessed using Bioanalyzer 2100 and RNA 1000 Nano LabChip Kit (Agilent, Santa Clara, CA, USA) with RNA integrity number >7.0. Poly(A). RNA was purified from total RNA (5 μg) using poly-T oligo-attached magnetic beads using two rounds of purification. Following purification, the mRNA was fragmented into small pieces using divalent cations under elevated temperatures. The fragmented RNA was then reverse-transcribed to construct the final cDNA library following the protocol of the mRNA Seq sample preparation kit (Illumina, San Diego, CA, USA). The average insert size for the paired-end libraries was 300 bp (±50 bp). Paired-end sequencing was performed on an Illumina Novaseq™ 6000 platform at LC Sciences (Hangzhou, China) in accordance with the manufacturer’s recommended protocol.

The genome and annotation of Stout camphor (*C. kanehirae*) were downloaded from NCBI [46]. Contamination from adaptor sequences was removed using Scythe v0.95 (https://github.com/vsbuffalo/scythe, accessed on 20 July 2023), and reads were trimmed using Sickle v1.33 (https://github.com/najoshi/sickle; accessed on 21 July 2023) with default settings. The cleaned reads were subsequently aligned to the *C. kanehirae* reference genome using TopHat v2.0.6 [47]. Read counts were generated using HTSeq v0.5.4p1 [47]. All statistical analyses were performed using R packages unless otherwise specified.

### 4.4. Analysis of the Differentially Expressed Unigenes

StringTie v2.2.1 was used to determine expression levels for mRNA by calculating FPKM [48]. Differential expression analysis was performed to identify differentially expressed genes (DEGs) using the following criteria: |log2(fold change)| > 1, *p*-value < 0.05, and FDR-adjusted *p*-value (padj) threshold of padj < 0.05. Functional annotation of the DEGs was conducted through GO and KEGG enrichment analyses using in-house Perl scripts.

Genes involved in the terpenoid backbone biosynthesis, monoterpenoid biosynthesis, sesquiterpenoid and triterpenoid biosynthesis, and diterpenoid biosynthesis pathways were identified based on annotation results. For the TPS family, two Pfam domains, PF03936 and PF01397, were used to identify against the proteomes using HMMER (version 3.0; cut-off at e < 10^−5^) [49]. Sequence lengths shorter than 200 amino acids were excluded from further analysis [46]. All identified genes and gene family members related to terpenoid biosynthesis were assessed and curated manually using BLAST 2.14.0 against NCBI and Uniprot databases. Putative or annotated protein sequences of TPS (*n* = 305) were aligned using MAGA7 [50]. A maximum likelihood phylogenetic tree was constructed and visualized using the EvolView v2 platform (http://www.evolgenius.info/evolview/#login; accessed on 6 August 2024). For the SDR family, the domain PF13567 was used to identify the proteomes using HMMER, as described above. The identified *SDR* genes were further classified using the InterPro database (https://www.ebi.ac.uk/interpro/; accessed on 10 August 2024). Members of the SDR110C subfamily were selected for motif analysis using MEGA7 and the MEME Suite v5.5.3 (http://meme-suite.org/; accessed on 10 August 2024). The TF gene family members were retrieved and analyzed in (https://planttfdb.gao-lab.org/; accessed on 17 August 2024).

### 4.5. RT-qPCR Analysis

Total RNA of ten individuals belonging to two chemotypes was extracted as described above, and RT-qPCR of related genes was performed using a PrimeScript RT Reagent Kit and SYBR Premix EX Taq Kit (TaKaRa, Kyoto, Japan) on CFX96™ Real Time detection system (Bio-Rad, Hercules, CA, USA), according to the manufacturer’s instructions. Normalized expression values of detected genes were calculated using CFX96™ data manager using *Actin* as a reference gene. The primers of target genes and the reference gene are listed in Table S11, and the reactions were performed as described above.

### 4.6. Statistical Analyses

All the experiments were performed with three replicates, and the results were presented as mean ± SD. The statistical analyses were performed using Student’s *t*-test in SPSS (version 17.0, IBM Corporation, Chicago, IL, USA).

## 5. Conclusions

In this study, metabolic and transcriptomic analyses of leaves were compared between linalool- and camphor-chemotypes of *C. camphora*. A comprehensive overview of terpenoid biosynthesis was presented in *C. camphora*. Monoterpenes were identified as the major components in the leaves of two chemotypes, with linalool and camphor being the most abundant components in the linalool chemotype and camphor chemotype, respectively. Transcriptome comparison between the two chemotypes identified 6499 DEGs, and KEGG enrichment analysis revealed that monoterpenoid and diterpenoid biosynthesis might contribute to the differential accumulation of terpenoids between the two chemotypes in *C. camphora*. A total of 65 genes were involved in terpenoid backbone biosynthesis, most of which were not differentially expressed between the two chemotypes, except for four *DXS* genes, one *HMGR* gene, and one *GPPS* gene. Among the 83 *CcTPS* genes, 42 were annotated as monoterpenoid synthesis enzymes, of which 27 monoterpenoid-related genes were differentially expressed between two chemotypes, and 17 genes were upregulated in the camphor type compared to the linalool type.

## Data Availability

The original contributions presented in the study are publicly available. These data can be found here: https://www.ncbi.nlm.nih.gov/sra (accessed on 11 February 2025), BioProject accession number PRJNA1222125.

## Supplementary Materials

The following supporting information can be downloaded at: https://www.mdpi.com/article/10.3390/plants14070991/s1, Figure S1: The statistics for the genomic regions of mapped reads; Figure S2: The PCA and pearson correlation analysis between samples; Figure S3: The GO and KEGG enrichment analysis for the DEGs between two chemotypes; Figure S4: Multiple alignment of deduced amino acid sequences; Figure S5: RT-qPCR expression patterns of candidate genes with their FPKM values in RNA-seq data; Table S1: The components of EO in two chemotypes of *C. camphora*; Table S2: Statistics of *C. camphora* transcriptomes used in this study; Table S3: Differentially expressed genes in two chemotypes of *C. camphora*; Table S4: The GO enrichment analysis; Table S5: The KEGG enrichment analysis; Table S6: Genes related to terpenoid biosynthesis in *C. camphora*; Table S7: Identification of genes involved in terpenoid backbone biosynthesis; Table S8: The basic information for *TPS* genes; Table S9: Numbers of TPS subfamilies in the six plant species; Table S10: The basic information for *SDR* genes; Table S11: Primers used for RT-qPCR analysis of selected genes.

## Abbreviations

The following abbreviations are used in this manuscript:

EO(s)	Essential oil(s).
TPS(s)	Terpene synthase(s).
mTPS(s)	Monoterpene synthase(s).
sTPS(s)	Sesquiterpene synthase(s).
GPP	Geranyl diphosphate.
NPP	Neryl diphosphate.
LiLINS	L. × intermedia linalool synthase.
LiBDH	L. × intermedia borneol dehydrogenase.
SDR	Short-chain dehydrogenase/reductase.
