# Supplementary material for: The Mining of Candidate Genes Involved in the Camphor Biosynthesis Pathway of Cinnamomum camphora
Source: Plants (Basel). 2025 Mar 21;14(7):991. doi: 10.3390/plants14070991 (PMC11990527; doi:10.3390/plants14070991)
Supplement: Supplementary file 1 [file plants-14-00991-s001.zip › Table S1 The components of essential oil in two chemotypes of C. camphora.pdf]

Table S1 The components of essential oil in two chemotypes of *C. camphora*

Number	RT	Name	Chemical formula	CAS	Relative content of camphor-type EO (%)	Relative content of linalool-type EO (%)
1	7.59	β-Thujene	$C_{10}H_{16}$	28634-89-1	0.25±0.07	0.10±0.25
2	7.83	α-Pinene	$C_{10}H_{16}$	7785-70-8	2.96±0.53	0.16±2.96
3	8.39	Camphene	$C_{10}H_{16}$	79-92-5	1.54±0.24	0.12±1.54
4	9.39	Sabinene	$C_{10}H_{16}$	3387-41-5	0.24±0.08	0.06±0.24
5	9.49	β-Pinene	$C_{10}H_{16}$	127-91-3	1.02±0.20	0.11±1.02
6	10.9	α-Ocimene	$C_{10}H_{16}$	502-99-8	0.01±0.01	--
7	10.11	β-Myrcene	$C_{10}H_{16}$	123-35-3	1.06±0.26	0.08±1.06
8	10.86	3-Carene	$C_{10}H_{16}$	13466-78-9	0.02±0.01	--
9	10.63	α-Phellandrene	$C_{10}H_{16}$	99-83-2	0.35±0.06	0.02±0.35
10	11.15	α-Terpinene	$C_{10}H_{16}$	99-86-5	0.09±0.02	0.03±0.09
11	11.57	o-Cymene	$C_{10}H_{14}$	527-84-4	0.20±0.06	--
12	11.64	Limonene	$C_{10}H_{16}$	138-86-3	3.01±0.74	0.10±3.01
13	11.88	Eucalyptol	$C_{10}H_{18}O$	470-82-6	0.76±0.21	0.21±0.76
14	12.16	(-)-α-Pinene	$C_{10}H_{16}$	7785-26-4	0.02±0.01	0.07±0.02
15	13.02	γ-Terpinene	$C_{10}H_{16}$	99-85-4	0.18±0.04	0.02±0.18
16	13.45	trans-4-Thujanol	$C_{10}H_{18}O$	17699-16-0	--	0.01±0.00
17	14.33	Terpinolene	$C_{10}H_{16}$	586-62-9	0.30±0.07	0.02±0.30
18	14.45	(E)-Linalol furanoxide	$C_{10}H_{18}O_2$	34995-77-2	0.01±0.01	0.21±0.01
19	14.88	Terpineol	$C_{10}H_{18}O$	7299-41-4	--	0.01±0.00
20	14.96	Linalool	$C_{10}H_{18}O$	78-70-6	0.34±0.18	75.97±0.34
21	15.23	Hotrienol	$C_{10}H_{18}O$	29957-43-5	--	0.27±0.00
22	15.91	p-Menth-2-en-1-ol	$C_{10}H_{18}O$	619-62-5	0.03±0.00	0.05±0.03
23	17.13	(+)-Camphor	$C_{10}H_{18}O$	464-49-3	62.85±4.49	0.1±0.00
24	17.99	Borneol	$C_{10}H_{18}O$	507-70-0	0.54±0.02	0.05±0.54
25	18.08	δ-Terpineol	$C_{10}H_{18}O$	7299-42-5	--	0.01±0.00
26	18.47	Terpinen-4-ol	$C_{10}H_{18}O$	562-74-3	0.64±0.11	0.09±0.64
27	19.17	α-Terpineol	$C_{10}H_{18}O$	98-55-5	0.89±0.17	0.1±0.89
28	20.87	Nerol	$C_{10}H_{18}O$	106-25-2	--	0.02±0.00
29	20.94	Citronellol	$C_{10}H_{20}O$	106-22-9	0.01±0.01	--
30	22.09	Geraniol	$C_{10}H_{18}O$	106-24-1	--	0.01±0.00
31	22.87	α-Citral	$C_{10}H_{18}O$	141-27-5	--	0.03±0.00
32	23.38	Bornyl acetate	$C_{12}H_{20}O_2$	76-49-3	0.21±0.05	0.06±0.21
33	25.42	δ-Elemene	$C_{15}H_{24}$	20307-84-0	0.10±0.04	0.03±0.1
34	25.93	α-Copaene	$C_{15}H_{24}$	3856-25-5	--	0.05±0.00
35	26.86	Ylangene	$C_{15}H_{24}$	14912-44-8	0.06±0.01	0.11±0.06
36	27.06	α-Cubebene	$C_{15}H_{24}$	17699-14-8	0.01±0.01	0.02±0.01

37	27.45	(-)-β-Bourbonene	$C_{15}H_{24}$	5208-59-3	0.05±0.01	0.08±0.05
38	27.79	β-Elemen	$C_{15}H_{24}$	515-13-9	0.52±0.17	0.36±0.52
39	28.92	Caryophyllene	$C_{15}H_{24}$	87-44-5	3.37±0.81	4.33±3.37
40	29.32	β-cubebene	$C_{15}H_{24}$	13744-15-5	0.03±0.00	0.02±0.03
41	29.54	γ-Elemene	$C_{15}H_{24}$	29873-99-2	0.13±0.06	0.1±0.13
42	29.93	(-)-Aristolene	$C_{15}H_{24}$	6831-16-9	0.07±0.02	0.08±0.07
43	30.35	Humulene	$C_{15}H_{24}$	6753-98-6	1.59±0.49	0.42±1.59
44	31.33	α-Amorphene	$C_{15}H_{24}$	483-75-0	0.05±0.02	0.04±0.05
45	31.5	Germacrene D	$C_{15}H_{24}$	23986-74-5	2.51±0.86	2.40±2.51
46	31.7	Selinene	$C_{15}H_{24}$	473-13-2	2.07±0.71	1.43±2.07
47	32.06	α-Guaiene	$C_{15}H_{24}$	3691-12-1	0.82±0.29	0.55±0.82
48	32.14	Bicyclogermacren	$C_{15}H_{24}$	24703-35-3	0.76±0.24	--
49	32.59	α-Amorphene	$C_{15}H_{24}$	483-75-0	0.03±0.01	0.03±0.03
50	32.98	(-)-α-Panasinsen	$C_{15}H_{24}$	56633-28-4	0.04±0.02	0.04±0.04
51	33.22	δ-Cadinene	$C_{15}H_{24}$	483-76-1	0.08±0.03	0.06±0.08
52	33.89	Dehydronerolidol	$C_{15}H_{24}O$	2387-68-0	0.01±0.01	0.03±0.01
53	34.33	Elemol	$C_{15}H_{26}O$	639-99-6	0.02±0.01	0.11±0.02
54	34.53	Germacrene B	$C_{15}H_{24}$	14523-57-1	0.76±0.28	0.74±0.76
55	34.84	Nerolidol	$C_{15}H_{26}O$	142-50-7	0.06±0.02	0.09±0.06
56	34.98	E-Nerolidol	$C_{15}H_{26}O$	40716-66-3	0.01±0.01	--
57	35.46	(-)-Spathulenol	$C_{15}H_{24}O$	77171-55-2	0.05±0.02	--
58	35.63	(-)-Globulol	$C_{15}H_{26}O$	489-41-8	0.04±0.03	0.31±0.04
59	35.98	Viridiflorol	$C_{15}H_{24}O$	552-02-3	0.01±0.01	0.01±0.01
60	36.21	1(5)-Guaien-11-ol	$C_{15}H_{24}O$	13822-35-0	0.02±0.02	0.03±0.02
61	36.68	Caryophyllene oxide	$C_{15}H_{24}O$	1139-30-6	0.01±0.01	--
62	36.78	Patchoulane	$C_{15}H_{26}$	25491-20-7	0.03±0.03	0.05±0.03
63	37.07	Shyobunol	$C_{15}H_{26}O$	35727-45-8	0.01±0.02	0.03±0.01
64	37.43	(+)-Spathulenol	$C_{15}H_{24}O$	6750-60-3	0.02±0.01	0.04±0.02
65	37.77	Isospathulenol	$C_{15}H_{24}O$	88395-46-4	0.01±0.01	--
66	38.21	Cubenol	$C_{15}H_{26}O$	21284-22-0	0.02±0.02	0.04±0.02
67	38.37	τ-Muurolol	$C_{15}H_{24}O$	19912-62-0	0.11±0.06	0.05±0.11
