# Supplementary material for: The Mining of Candidate Genes Involved in the Camphor Biosynthesis Pathway of Cinnamomum camphora
Source: Plants (Basel). 2025 Mar 21;14(7):991. doi: 10.3390/plants14070991 (PMC11990527; doi:10.3390/plants14070991)
Supplement: Supplementary file 1 [file plants-14-00991-s001.zip › Table S2 Statistics of Cinnamomum camphora transcriptomes in this study.pdf]

Table S2 Statistics of *Cinnamomum camphora* transcriptomes in this study.

Sample	Raw reads	Clean reads	Clean read rate (%)	Mapping reads	Mapping rate (%)
Linalool 1	42544068	41596770	97.77	35859676	86.21
Linalool 2	48665906	47131082	96.85	40009798	84.89
Linalool 3	46739708	45538112	97.43	38808357	85.22
Camphor 1	46132790	43298476	93.86	37492402	86.59
Camphor 2	37135256	35924646	96.74	31246088	86.98
Camphor 3	38369334	37313410	97.25	32278670	86.51
