# Supplementary material for: The Mining of Candidate Genes Involved in the Camphor Biosynthesis Pathway of Cinnamomum camphora
Source: Plants (Basel). 2025 Mar 21;14(7):991. doi: 10.3390/plants14070991 (PMC11990527; doi:10.3390/plants14070991)
Supplement: Supplementary file 1 [file plants-14-00991-s001.zip › Table S6 Genes related to secondary metablolite biosynthesis in C. camphora.pdf]

Table S6 Genes related to terpenoid biosynthesis in *C. camphora*

Category	KEGG pathways (Ko)	Total gene number	Total Ko entries	DEGs	Ko entries
Metabolism of terpenoids and polyketides	Terpenoid backbone Biosynthesis (900)	65	27	16	7
	Monoterpenoid Biosynthesis (902)	43	8	33	5
	Sesquiterpenoid and triterpenoid Biosynthesis (909)	37	6	14	5
	Diterpenoid biosynthesis (904)	46	5	17	5
	Carotenoid biosynthesis (906)	64	18	16	7
	Ubiquinone and other terpenoid-quinone biosynthesis (130)	50	20	11	5
	Brassinosteroid biosynthesis (905)	20	7	4	6
	Zeatin biosynthesis (908)	20	7	8	5
Biosynthesis of other secondary metabolites	Phenylpropanoid biosynthesis (940)	176	14	62	10
	Stilbenoid, diarylheptanoid and gingerol biosynthesis (945)	49	5	18	5
	Flavone and flavonol biosynthesis (944)	14	6	2	2
	Anthocyanin biosynthesis (942)	8	3	3	2
	Isoflavonoid biosynthesis (943)	8	3	1	1
	Indole alkaloid biosynthesis (901)	44	2	20	2
	Isoquinoline alkaloid biosynthesis (950)	90	9	29	7
	Tropane, piperidine and pyridine alkaloid biosynthesis (960)	40	8	11	5
	Caffeine metabolism (232)	7	3	5	3
	Betalain biosynthesis (965)	37	2	14	1
	Glucosinolate biosynthesis (966)	25	3	0	0
Total		843	156	284	83
