# Supplementary material for: The Mining of Candidate Genes Involved in the Camphor Biosynthesis Pathway of Cinnamomum camphora
Source: Plants (Basel). 2025 Mar 21;14(7):991. doi: 10.3390/plants14070991 (PMC11990527; doi:10.3390/plants14070991)
Supplement: Supplementary file 1 [file plants-14-00991-s001.zip › Table S9 Numbers of TPS subfamilies in the six plant species.pdf]

Table S9 Numbers of TPS subfamilies in the six plant species

TPS subfamilies	a	b	c	d[a]	e/f	g	h[b]	Total no.
function species	C15	C10, IspS	CPS, C20	C10,C15,C20	KS,C20	C10	CPS, KS	
C. camphora	22	45	1	-	11	7	-	86
C. kanehirae	25	51	2	-	12	11	-	101
A. thaliana	23	5	1	-	2	1	-	32
O. sativa	17	-	3	-	10	1	-	31
S. moellendorffii	-	-	3	-	3	-	8	14
P. trichocarpa	13	13	2	-	3	2	-	33
A. grandis	0	0	0	11	0	0	0	11

[a]TPS-d subfamily is gymnosperm specific, [b]TPS-h subfamily is ferns specific. CPS, copalyl synthase; KS, kaurene synthase; IspS, isoprene synthases.
