# Supplementary material for: The Mining of Candidate Genes Involved in the Camphor Biosynthesis Pathway of Cinnamomum camphora
Source: Plants (Basel). 2025 Mar 21;14(7):991. doi: 10.3390/plants14070991 (PMC11990527; doi:10.3390/plants14070991)
Supplement: Supplementary file 1 [file plants-14-00991-s001.zip › Table S11 Primers.pdf]

Table S11 Primers used for qRT-PCR analysis of selected genes.

Gene name	Gene specific forward primer (5' to 3')	Gene specific reverse primer (5' to 3')
Cc_TPS10	GAAGCTGCCATTTACTCGGG	TGGAAGTTGTTGGATGGCAC
Cc_TPS20	GGTGGGATCGTAGCATGGTA	GCAAGTGATCAAGGCGACAT
Cc_TPS27	AGACCTCCTCAGCTCTCTCA	CTCAAGCCAAGTCCCAAAGC
Cc_TPS47	GGGAATGCTGAGCTTGTACG	ACGTCCATGTACCACCTAGC
Cc_TPS62	CGTTCACAAGCACGTACCTC	ACCAACTTGATGCCGTCCTA
Cc_TPS80	TCCTCCCAAATCCCAGACAC	TGCCTCTACCTTGTAGCCAC
Cc_SDR41	CTCACAAAGAATGCTGCCGT	CCAGGTAAAGAGCAGCCAGA
Cc_FAR1	TGCTACAGGTGAGAGTTGGG	ATTGTAAGCCCAACCGAGGA
Cc_NAC35	ATGCCAGATACATGCCCCTT	GCCAGTTCTCTTCCTCAGGT
Cc_WRKY70	AGCTACCCCTTATGACGACG	GTTGCCCCATGTATGTGACC
Cc_DXS1	ACCCCGTTAGAGATTGGCAA	CCTGATCAGCTCTGTGTCCA
Cc_DXS2	AAGGAGGTCGAGAATGCACA	CCAGATAGCCCGCATTGTTC
actin	TGTTCTGGACTCGGGTGA	ATGGATTCCTGCTGCTTC
