# Supplementary figures and images for: The Mining of Candidate Genes Involved in the Camphor Biosynthesis Pathway of Cinnamomum camphora
Source: Plants (Basel). 2025 Mar 21;14(7):991. doi: 10.3390/plants14070991 (PMC11990527; doi:10.3390/plants14070991)

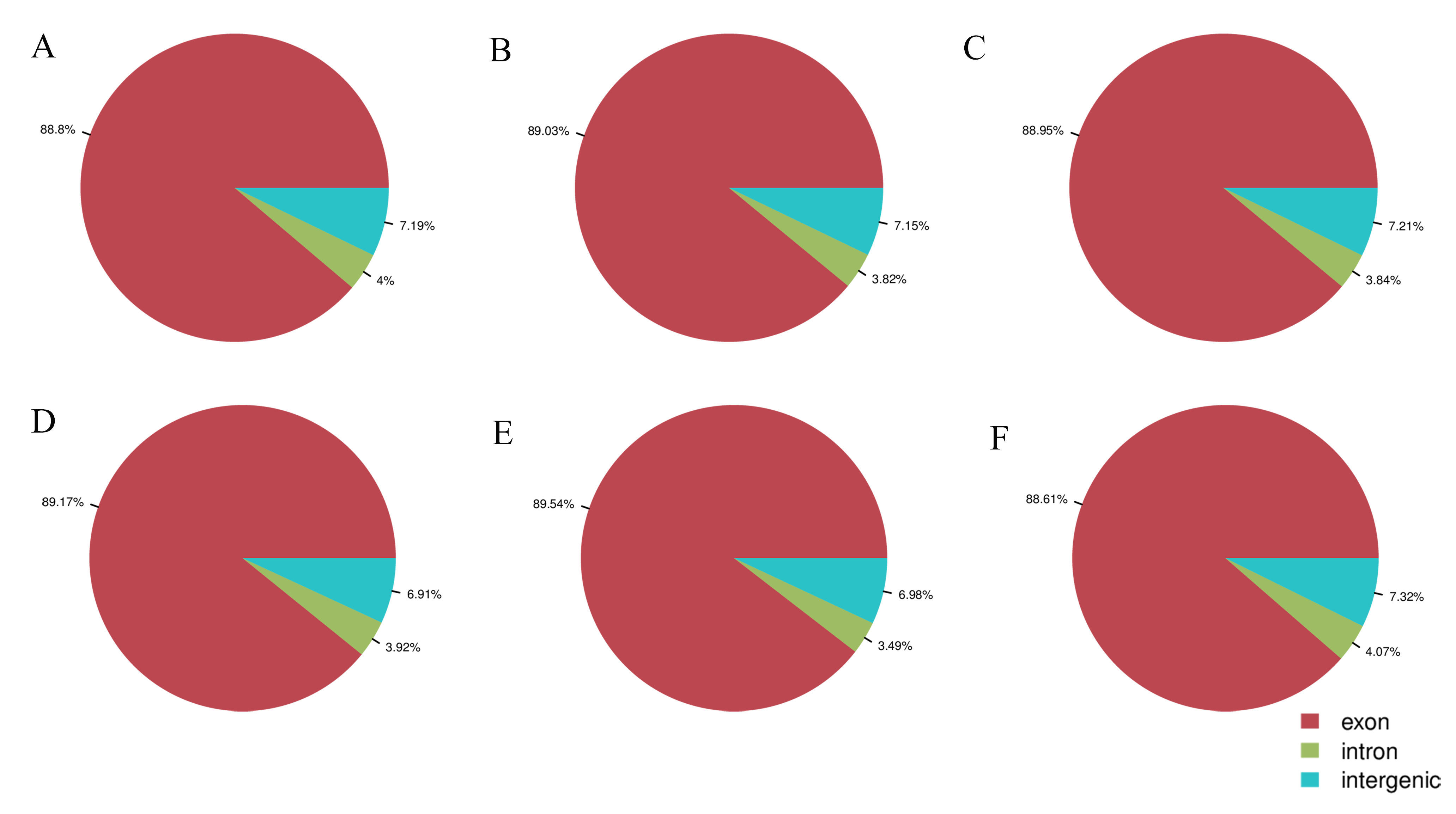

Supplement: Supplementary file 1 [file plants-14-00991-s001.zip › Figure S1.tif]

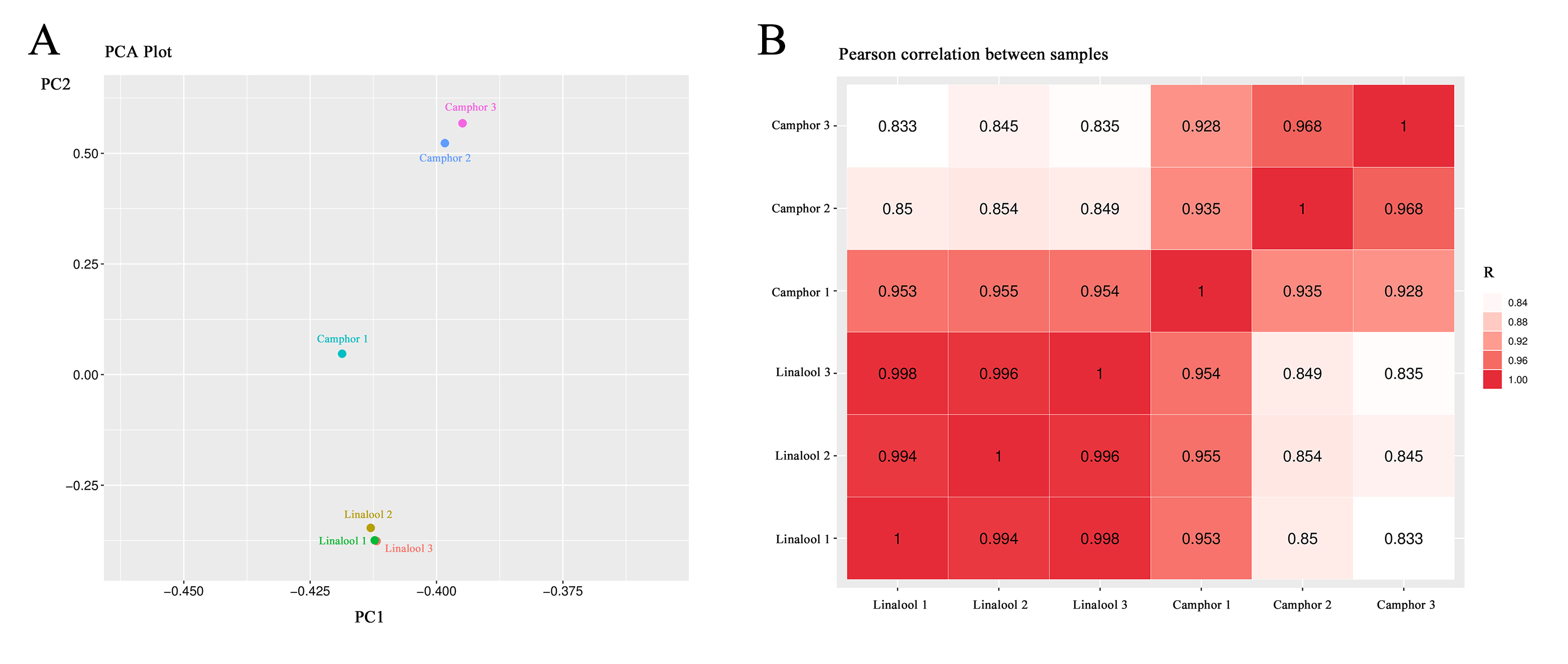

Supplement: Supplementary file 1 [file plants-14-00991-s001.zip › Figure S2.tif]

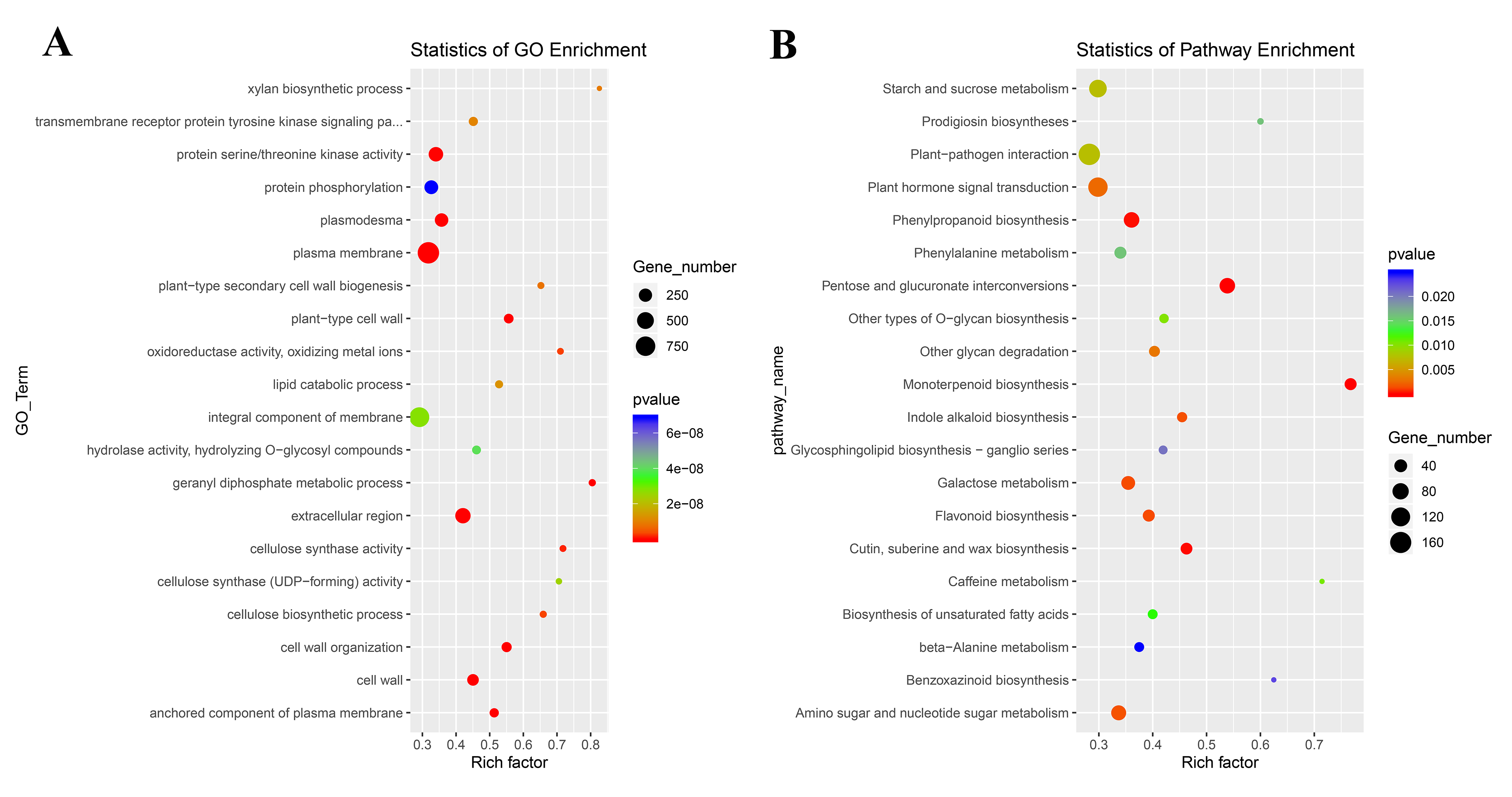

Supplement: Supplementary file 1 [file plants-14-00991-s001.zip › Figure S3.tif]

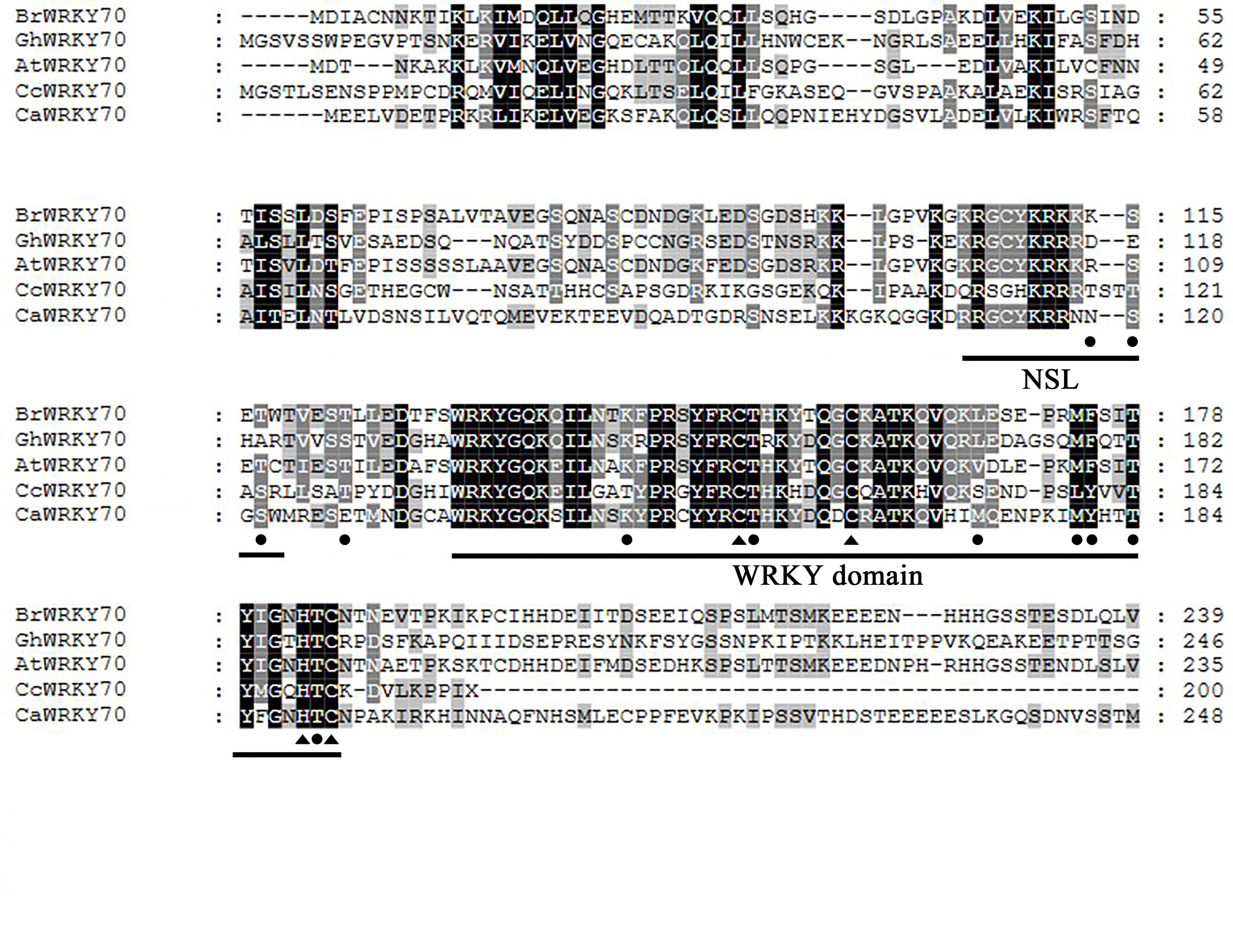

Supplement: Supplementary file 1 [file plants-14-00991-s001.zip › Figure S4.tif]

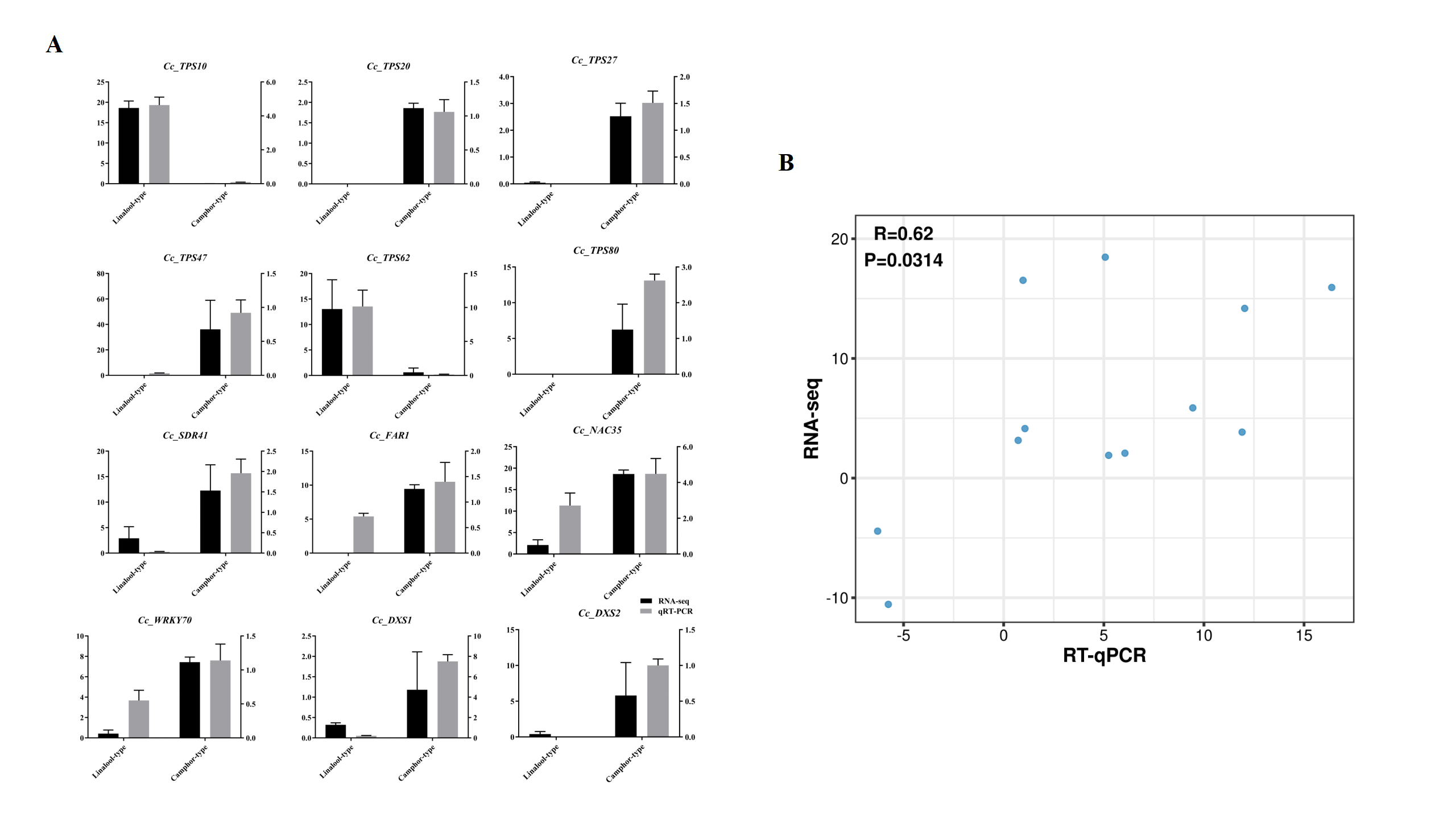

Supplement: Supplementary file 1 [file plants-14-00991-s001.zip › Figure S5.tif]
